# Designing a C_84_ fullerene as a specific voltage-gated sodium channel blocker

**DOI:** 10.1186/1556-276X-8-323

**Published:** 2013-07-16

**Authors:** Tamsyn A Hilder, Shin-Ho Chung

**Affiliations:** 1 Computational Biophysics Group, Research School of Biology, Australian National University, ACT 0200 Canberra, Australia

**Keywords:** C_84_ fullerene derivative, Bacterial voltage-gated sodium channel Na_v_Ab, Voltage-gated potassium channel Kv1.3, Binding, Molecular dynamics, 87.85.Qr, 87.85.Rs, 87.10.Tf

## Abstract

Fullerene derivatives demonstrate considerable potential for numerous biological applications, such as the effective inhibition of HIV protease. Recently, they were identified for their ability to indiscriminately block biological ion channels. A fullerene derivative which specifically blocks a particular ion channel could lead to a new set of drug leads for the treatment of various ion channel-related diseases. Here, we demonstrate their extraordinary potential by designing a fullerene which mimics some of the functions of μ-conotoxin, a peptide derived from cone snail venom which potently binds to the bacterial voltage-gated sodium channel (Na_v_Ab). We show, using molecular dynamics simulations, that the C_84_ fullerene with six lysine derivatives uniformly attached to its surface is selective to Na_v_Ab over a voltage-gated potassium channel (Kv1.3). The side chain of one of the lysine residues protrudes into the selectivity filter of the channel, while the methionine residues located just outside of the channel form hydrophobic contacts with the carbon atoms of the fullerene. The modified C_84_ fullerene strongly binds to the Na_v_Ab channel with an affinity of 46 nM but binds weakly to Kv1.3 with an affinity of 3 mM. This potent blocker of Na_v_Ab may serve as a structural template from which potent compounds can be designed for the targeting of mammalian Nav channels. There is a genuine need to target mammalian Nav channels as a form of treatment of various diseases which have been linked to their malfunction, such as epilepsy and chronic pain.

## Background

Typically, toxins from venomous species such as cone snails, spiders, and snakes are investigated as possible drug leads for ion channel blockers. Converting these toxins to drugs represents a considerable challenge [1]. For example, disulfide bridges in these peptides, abundant in all toxins, are vulnerable to scrambling and reduction in certain extracellular environments and therefore must be replaced [1-4]. Nanomaterials designed to mimic the main features of these complex toxin structures present exciting opportunities to specifically target a particular ion channel subtype and may alleviate some of the challenges of these peptides.

Increasing attention is being given to fullerenes for biological applications including antiviral and antibacterial agents, antioxidants, vectors for drug/gene delivery, photodynamic therapy, enzyme inhibitors, and diagnostics (e.g., magnetic resonance imaging) [5,6]. For example, fullerene derivatives have been shown to bind to and inhibit the activity of HIV protease [7]. Fullerenes consist of a hollow carbon cage structure formed by 20 to as many as 300 carbon atoms [8,9]. The most abundantly produced are those with 60 and 70 carbon atoms. Fullerenes are insoluble in aqueous solution and aggregate easily. Therefore, there has been significant work into making these structures soluble so that they can be utilized for their potential biomedical applications. One method which increases their solubility is chemical functionalization with moieties such as amino acids and carboxylic acid [5]. Fullerene chemistry has been intensely developed, and the main efforts are now devoted to broaden their application [6].

In 2003, Park et al. [10] identified non-functionalized carbon nanotubes and C_60_ fullerenes as a novel class of ion channel blockers. Their experiments on various biological ion channels demonstrated that these nanostructures indiscriminately interfere with the activity of potassium channels depending on their geometric structure and size. Similarly, experiments by Chhowalla et al. [11] and Xu et al. [12] demonstrated that functionalized multi-walled and single-walled carbon nanotubes affected the activity of ion channels in Chinese hamster ovary cells (CHO) and undifferentiated pheochromocytoma (PC12) cells, respectively. Park et al. [10] also examined the binding of their fullerenes and nanotubes to KcsA using docking simulations and proposed that the molecules block the entrance to the pore. In contrast, Kraszewski et al. [13] showed using molecular dynamics simulations that C_60_ fullerenes do not bind to the selectivity filter. Instead, they demonstrated that C_60_ fullerenes bind strongly to the hydrophobic residues of the extracellular loops in the three potassium channels they examined, namely KcsA, MthK, and Kv1.2, and suggest that these fullerenes may hinder the function of potassium channels [13]. Similarly, Monticelli et al. examined the interaction of a C_70_ fullerene with the Kv1.2 potassium channel using molecular dynamics and found that they made contact with hydrophobic residues in the extracellular or intracellular loops, but not the selectivity filter [14]. They also examined C_70_ fullerenes fully coated in gallic acid to stabilize the fullerenes in solution. These gallic acid coated fullerenes were also shown to make contact with the extracellular or intracellular loops, but not the selectivity filter [14]. Monticelli and co-workers [14,15] have also shown using molecular dynamics that non-functionalized fullerenes agglomerate within the hydrophobic layer of lipid bilayers.

**Figure 1:**
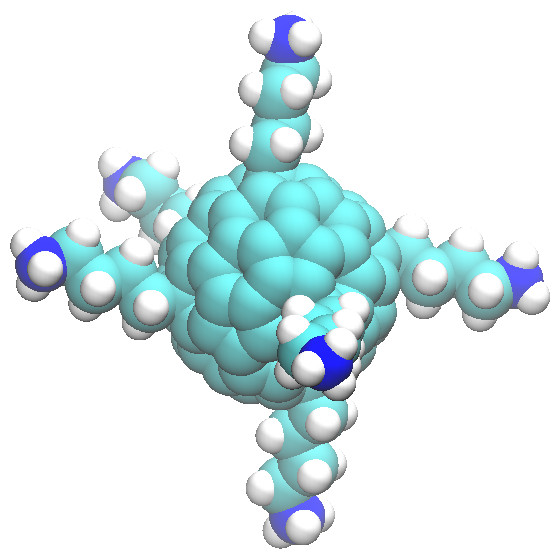
**[Lys]-fullerene structure.** Optimized structure of the [Lys]-fullerene.

In this paper, we design a fullerene to mimic the structure of μ-conotoxin, which has been shown to bind with strong affinity to Na_v_Ab [16,17]. Our fullerene molecule, illustrated in Figure 1, contains 84 carbon atoms and has six lysine derivatives uniformly attached to its surface. In essence, the C_84_ fullerene cage mimics the rigid globular structure of the μ-conotoxin molecule, and the lysine derivatives mimic the flexible positively charged arms of μ-conotoxin which are shown to bind to the channel and within the selectivity filter of Na_v_Ab [16]. By comparing the binding of the C_84_ fullerene derivative to two membrane ion channels, the voltage-gated potassium channel Kv1.3 and the bacterial voltage-gated sodium channel Na_v_Ab, we are able to demonstrate its specificity to Na_v_Ab. Kv1.3 is a mammalian voltage-gated potassium channel, whereas Na_v_Ab is a voltage-gated sodium channel present in bacteria. There is a genuine need to target mammalian voltage-gated sodium channels as a form of treatment of various diseases which have been linked to their malfunction, such as epilepsy, neuropathic pain, and long QT syndrome [18-20]. This work suggests the possibility of fullerene derivatives as possible drug leads for the treatment of these diseases. Alternatively, although the function of bacterial voltage-gated sodium channels is relatively unknown, it has been proposed that they may play a role in flagella mobility [21]. The flagellum generates chemotaxis which is critical for the cell's survival [22], as chemotaxis is one of the sensing systems that bacteria have to monitor and respond to changes in their environment [23]. Therefore, these fullerene derivatives may also have potential as antibacterial agents.

## Methods

Although C_60_ and C_70_ fullerenes are the most abundantly produced in carbon soot, higher fullerenes such as C_76_, C_78_, and C_84_ have also been isolated [24,25] and are among the most abundant higher fullerenes [26]. We generate an initial C_84_ fullerene molecule using the fullerene library available in the Nanotube Modeler 1.7.3 software [27]. The C_84_ fullerene has six favorable isomers [28], and of these, the D_2_ and D_2d_ have the lowest energy [29]. We choose the structure with D_2d_ symmetry (structure number 23 in Nanotube Modeler) as this has also been reported as the most commonly observed in experiments [28]. The C_84_ fullerene has an approximate diameter of 8 Å.

Ideally, an ion channel blocker design would have flexible side chains which can bind to the channel and block the entrance to the pore. The D_2d_ isomer of C_84_ has been shown to have the most localized *π* bonding of the fullerenes that have been isolated and has therefore been suggested as being the most reactive toward addition reactions [28]. Researchers [30-32] have also shown that it is possible to attach various chemical species to the outside of fullerene molecules. For example, phenylalanine and lysine amino acid derivatives have been attached to the C_60_ fullerene [30,31]. Therefore, we import the C_84_ fullerene structure into ArgusLab 4.0.1 and attach six lysine derivatives to its outside surface [33]. A similar water-soluble amino-fullerene derivative with five cysteine moieties attached to the surface of C_60_ fullerene has previously been synthesized and characterized by Hu et al. [34]. They demonstrated the ability of this fullerene derivative to prevent oxidative-induced cell death without evident toxicity [34]. We choose positively charged residues with the aim of mimicking the function of μ-conotoxin to Na_v_Ab. The distance between nitrogen atoms on opposing lysine chains is approximately 21 Å.

The modified fullerene (C_84_(C_4_H_8_NH_3_^+^)_6_ structure is optimized in ArgusLab [33] and is shown in Figure 1. The geometry optimizations were performed using default parameters, the Broyden-Fletcher-Goldfarb-Shanno algorithm and the universal force field. Restricted Hartree-Fock method was used, where the molecule is a closed shell system with all orbitals doubly occupied. All optimization processes are performed until the Hartree-Fock self-consistent field converged to 10^−10^ kcal/mol and the gradient converged to 10^−1^ kcal/mol/Å. Throughout this paper, this modified C_84_ fullerene is referred to as [Lys]-fullerene.

The coordinates of Na_v_Ab are obtained from the protein database [PDB:3RVY] [35]. We obtain a homology model of Kv1.3 using the refined structure of the Kv1.2 channel (PDB:SLUT) as a template [36]. The generation of the homology model for Kv1.3 is described in detail in Chen et al. [37], where it was shown to reproduce experimental conduction properties and binding affinities to charybdotoxin and ShK. We chose to examine the binding of the [Lys]-fullerene to Kv1.3, giving us the opportunity to directly compare our results with the binding of polypeptide toxins [37,38].

Molecular dynamics (MD) simulations are used to determine the bound configuration of the [Lys]-fullerene and calculate the potential of mean force (PMF) of the [Lys]-fullerene binding to the channel. All MD simulations are performed using NAMD 2.8 and visualized using VMD 1.9 [39,40]. Throughout, we use the CHARMM36 force field [41,42] and TIP3P water, with a time step of 2 fs, at constant pressure (1 atm), and temperature (300 K). The channel and fullerene complex are embedded in a POPC lipid bilayer, solvated in approximately a 100 × 100 × 100 Å^3^ box of water. Potassium/sodium (for Kv1.3/Na_v_Ab) and chloride ions are added to both neutralize the system and simulate a 250-mM ionic concentration. The protein is initially held fixed to allow the water and ions to equilibrate during the simulation period of 0.1 ns, and in subsequent simulations, the protein and lipid bilayer center of mass is held by a harmonic constraint of 0.2 kcal/mol/Å^2^. A similar methodology has been used to investigate the binding of toxins to ion channels [16,37,43].

The [Lys]-fullerene is initially placed near the entrance of the selectivity filter (at *z* = 22 Å) and the system is allowed to equilibrate for 1 to 3 ns with the fullerene unconstrained. The PMF for the binding of the [Lys]-fullerene to the Na_v_Ab and Kv1.3 channels is determined using umbrella sampling with this equilibrated structure. Umbrella sampling windows are generated using steered MD simulations with a force of 30 kcal/mol/Å applied to pull the fullerene out of the binding site. During the steered MD simulations the backbone atoms of the protein are held fixed and the atoms of the fullerene are held by a harmonic constraint of 0.2 kcal/mol/Å^2^ to maintain the root-mean-square deviation, with reference to a starting configuration below 0.25 Å so that no significant distortion takes place. The channel central axis (*z*-axis) is used as the reaction coordinate. Pulling generates a continuous number of configurations along the permeation pathway so that umbrella sampling windows can be constructed every 0.5 Å.

During umbrella sampling the center of mass of the backbone atoms of the fullerene is confined to be within a cylinder of 8 and 13 Å centered on the channel axis for Kv1.3 and Na_v_Ab, respectively, and beyond this, a harmonic potential of 20 kcal/mol/Å^2^ is applied. These values are shown to provide adequate sampling. Moreover, a force constant of 30 kcal/mol/Å^2^ is applied in the *z* direction to constrain the center of mass of fullerene to the sampling window. The center of mass coordinates of the backbone atoms of the fullerene is saved every 0.5 ps. The PMF is then constructed along the *z* direction using the weighted histogram analysis method [44] and the center of mass coordinates. Each sampling window is run for 5 ns. The PMF is shown to converge as the depth changes by less than 0.5 kT when simulations are run for a further 1 ns.

The dissociation constant (*K*_d_) in the unit of molar is estimated to be [37,45,46]:

(1) $$K_{d}^{-1}=1,000\;N_{A}\pi R^{2}\int_{z1}^{z2}\exp\left(-W\left(z\right)/k_{B}T\right)\mathrm{dz},$$

where *W(z)* is the 1D PMF with the zero point located at the bulk, 1,000 *N*_A_ is used to convert from cubic meter to liter per mole, *k*_B_ and *T* are Boltzmann's constant and temperature, respectively, *z*_1_ is in the binding pocket, and *z*_2_ is in the bulk [46]. Although Equation 1 was originally derived for the binding of an ion to a channel [45], it has also been successfully applied to toxin binding [16,37,43]. Note that the windows at 38.5 and 42.0 Å for Na_v_Ab and Kv1.3, respectively, are assumed to be bulk, and the PMF is therefore set to zero at this *z* position. The fullerene is docked to Na_v_Ab and Kv1.3 at *z* = 20.5 Å and *z* = 23.0 Å, respectively, and the center of mass is located at *z* = 0 Å. A hydrogen bond is assumed to be formed if the donor-acceptor distance is within 3.0 Å and the donor-hydrogen-acceptor angle is ≥150. A salt bridge is formed between the fullerene and ion channel if the distance between any of the nitrogen atoms on the fullerene side chains and the oxygen atoms of an acidic residue on the ion channel is <4 Å.

## Results and discussion

**Figure 2:**
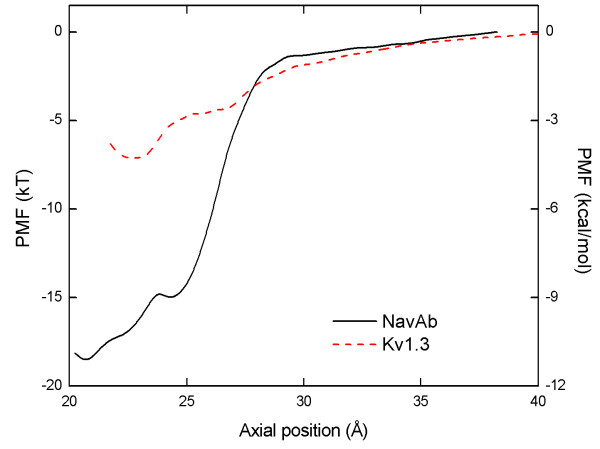
**Potential of mean force (PMF).** PMF for the cleavage of [Lys]-fullerene from the Na_v_Ab and Kv1.3 channels.

Figure 2 illustrates the PMF for the cleavage of [Lys]-fullerene from the Na_v_Ab and Kv1.3 channels. The axial position in Figure 2 is measured from the center of mass of the channel to that of the [Lys]-fullerene. The PMF reaches a minimum at 20.5 Å for Na_v_Ab, with a well depth of −18.7 kT. For Kv1.3, the PMF reaches a minimum at 23.0 Å, with a well depth of −7.1 kT. We find that the binding between [Lys]-fullerene and both Na_v_Ab and Kv1.3 is stable and so all 5 ns of umbrella sampling is used. It is assumed that the properties for the window at 38.5 and 42.0 Å for Na_v_Ab and Kv1.3, respectively, are similar to those in bulk, and therefore, the PMF is set to 0 at this point.

Using Equation 1, we obtain dissociation constants, *K*_d_, of 46 nM and 3 mM for the Na_v_Ab and Kv1.3 channels, respectively. In comparison, in MD simulations, Chen and Chung [16] found that μ-conotoxin binds to Na_v_Ab with a well depth of approximately −25 kT and a binding affinity of 0.1 nM. French and colleagues [17] have recently confirmed this result experimentally and obtained a binding affinity of 0.005 nM for the NaChBac, a bacterial channel closely related to Na_v_Ab. This [Lys]-fullerene mimic of μ-conotoxin is specific to Na_v_Ab over Kv1.3 and presents exciting opportunities for future drug development research.

**Figure 3:**
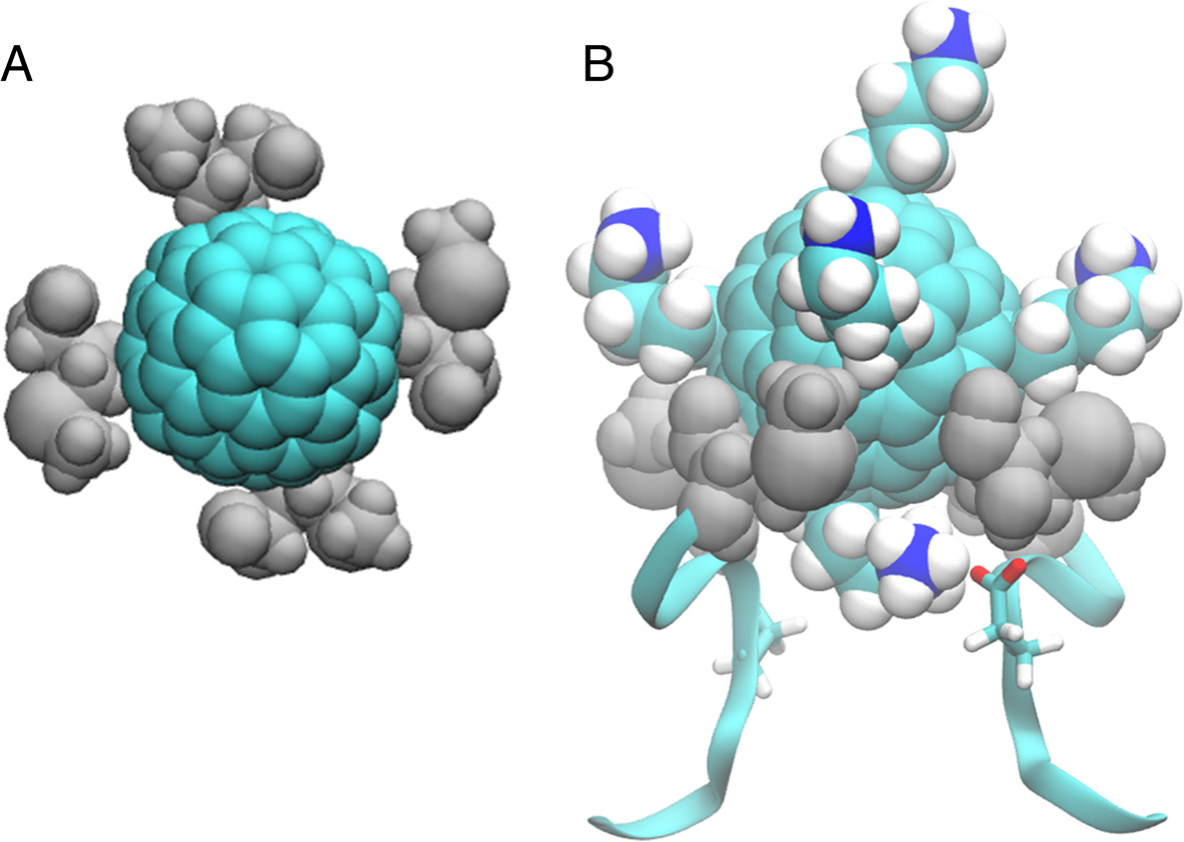
**Binding of [Lys]-fullerene to the outer vestibule of Na**_**v**_**Ab. (A)** Top view illustrating the four Met181 residues (shown in grey) coordinating the [Lys]-fullerene molecule. Note that the lysine side chains of the [Lys]-fullerene have been removed for clarity. **(B)** Side view illustrating the Met181 residues and Glu177 interaction with one of the lysine chains of the [Lys]-fullerene.

To characterize the interactions between the [Lys]-fullerene and the two channels, we examine the umbrella sampling window located at the minimum of the PMF, 20.5 and 23.0 Å in Na_v_Ab and Kv1.3, respectively. Examining the docked structure to Na_v_Ab more closely, we find that four methionine residues near the entrance to the pore (located at position 181) form a hydrophobic interaction with the fullerene surface, ‘coordinating’ the blocker to the pore, as illustrated in Figure 3A. No water molecules are present between the Met181 residues and the fullerene surface. Moreover, one of the lysine side chains of the [Lys]-fullerene is protruding into the selectivity filter. In Na_v_Ab, this side chain binds to the glutamate residue at position 177 (as shown in Figure 3B) with an average of 0.9 ± 0.6 hydrogen bonds. Glu177 has previously been identified as a blocking site for tetrodoxins and saxotoxins, and aligns with the glutamate residues that determine selectivity in Nav and Cav channels [35] (illustrated in the sequence alignment in Table 1). At approximately 24.5 Å, the [Lys]-fullerene sits off the center relative to the selectivity filter, bound to only two of the four Met181 residues. Moreover, the lysine derivative of the [Lys]-fullerene is no longer occluding the pore at this distance, allowing an open pore to occur. As the [Lys]-fullerene moves away from the pore entrance, the Met181 residues rotate so as to maximize the hydrophobic interaction until this interaction is completely cleaved when the [Lys]-fullerene reaches a distance of approximately 27.5 Å. The hydrophobic interaction between the Met181 residues and the fullerene surface is the main cause of the strong binding to the Na_v_Ab channel. In density functional calculations, the free energy of dissociation of methionine from a C_60_ fullerene is −12.121 kcal/mol [47].

**Table 1:** **Sequence alignment between Kv1.3, Na**_**v**_**Ab, and Nav1.8**

	**Sequence alignment**											
Kv1.3	V	V	T	M	T	T	V	G	Y	G	D	M^a^
Na_v_Ab	F	Q	V	M	T	L	E^b^	S	W	S	M^a^	G
Nav1.8 I	F	R	L	M	T	Q	D^b^	S	W	E	R	L^a^
Nav1.8 II	F	R	I	L	C	G	E^b^	W	I	E	N	M^a^
Nav1.8 III	L	Q	V	A	T	F	K^b^	G	W	M	D	I^a^
Nav1.8 IV	F	Q	I	T	T	S	A^b^	G	W	D	G	L^a^

Kv1.3 and Na_v_Ab are homotetramers, and Nav1.8 is a heterotetramer. ^b^Homologues to Glu177. ^a^Possible homologues to Met181.

**Figure 4:**
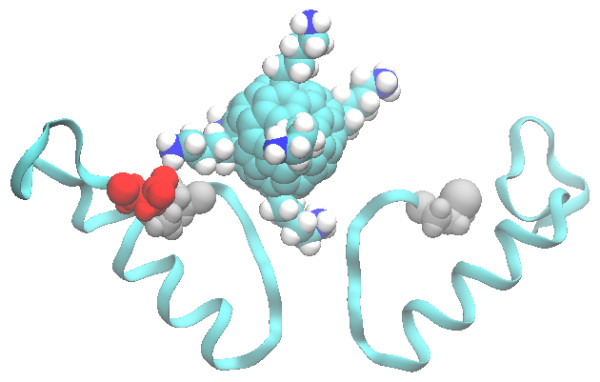
**Side view of the binding of [Lys]-fullerene to the outer vestibule of Kv1.3.** The Glu420 residue on chain A is shown in red, and the Met450 residues are shown in grey.

Similarly, by examining the docked structure to Kv1.3, we observe that one of the lysine side chains of the [Lys]-fullerene is protruding into the selectivity filter, as shown in Figure 4, with an average of 0.5 ± 0.8 hydrogen bonds. In some of the trajectories, one other lysine side chain makes contact with a glutamate residue on the outer vestibule at position 420 (shown as red in Figure 4), but over the entire simulation, there is only an average of 0.08 ± 0.3 hydrogen bonds between these two residues. Therefore, it is unlikely that this contact is stable. There is a methionine (Met450) residue in a similar position to the Met181 residues of Na_v_Ab, as shown in the sequence alignment in Table 1. However, in Kv1.3, these methionine residues are acting to stabilize the channel and therefore cannot flip outwards towards the fullerene. In contrast to Na_v_Ab, these methionine residues are unable to form a hydrophobic interaction with the [Lys]-fullerene surface, as shown in Figure 4. Amino acid sequences of the Na_v_Ab and Kv1.3 ion channels were obtained from the National Center for Biotechnology Information (NCBI) protein database (NCBI:3RVY_A, NCBI:NP_002223.3, respectively) [35]. The sequences were aligned using multiple sequence comparison by log-expectation (MUSCLE) [48].

Bacterial and mammalian channels differ significantly in both sequence and structure. In an attempt to understand how the [Lys]-fullerene might bind to a mammalian Nav channel, we align the sequence of Na_v_Ab to Nav1.8. Although μ-conotoxin is sensitive to Nav1 channels, Nav1.8 is both tetrodotoxin and μ-conotoxin insensitive [19,49]. The Nav1.8 sequence has recently been studied for gain-of-function mutations which have been linked to painful peripheral neuropathy [50]. A few selective blockers of Nav1.8 have been identified, such as A-803467 and μO-conotoxin, and have been shown to suppress chronic pain behavior [19,20]. Therefore, it is interesting to consider the sensitivity of Nav1.8 to [Lys]-fullerene. Amino acid sequences of the Na_v_Ab and Nav1.8 ion channels were obtained from the NCBI protein database (NCBI:3RVY_A, NCBI:NP_006505.2, respectively) [35,50], and the sequences were aligned using MUSCLE [48]. A comparison of the two sequences, shown in Table 1, demonstrates that Glu177 in Na_v_Ab aligns with the Asp-Glu-Lys-Ala (DEKA) residues of the selectivity filter of Nav1.8. As mentioned, the four methionine residues at position 181 form hydrophobic bonds with the fullerene molecule ‘coordinating’ it to the pore of Na_v_Ab. In Nav1.8, there are four hydrophobic residues in a similar position to Met181 and in particular Leu-Met-Iso-Leu (LMIL). It may be possible that a similar hydrophobic bond could form between the fullerene and this mammalian Nav channel. However, in Kv1.3, the methionine residue does not contribute to the binding of [Lys]-fullerene and instead stabilizes the channel. A similar mechanism could occur in Nav1.8. Unfortunately, no crystal structure of Nav1.8 or any other mammalian Nav channel is currently available. Therefore, to confirm such a hypothesis requires significant future work such as building a Nav1.8 homology model and conducting molecular dynamics simulations to ascertain the binding affinity of the [Lys]-fullerene.

Carbon-based nanomaterials offer the possibility of some exciting applications, such as drug delivery, selective cell destructive agents, biosensors, and diagnostics. Further development is needed regarding the toxicity of these materials in both biological and environmental environments, in the short and long terms, for these applications to be brought into widespread use. We refer the reader to recent reviews on the use of carbon nanotubes and fullerenes in biology and medicine [5,6,51]. Typically, non-functionalized carbon-based nanomaterials are considered to be toxic, but significant work has been done to make these structures soluble and biocompatible. For example, it has been demonstrated that C_60_ fullerene with five cysteine residues attached to its surface is water soluble and does not cause cellular toxicity [34].

As with any drug lead, to move from an idea to a marketable drug can take between 10 to 15 years. Therefore, significant research effort is required to develop this theoretical [Lys]-fullerene design into a drug for therapeutic use. Future simulations are required to determine whether these compounds are potent blockers of mammalian Nav channels and if they are specific to a particular channel sub-type. Following this, experiments would need to be performed to confirm theoretical findings and determine toxicity profiles. Polypeptide toxins from venomous animals have evolved over millions of years, aimed at rapidly immobilizing and capturing prey. Since they act on a broad spectrum of ion channel families and are rapidly degraded *in vivo*, converting these toxins to drugs represents a considerable challenge, and attempts are being made to synthesize smaller and more durable mimetic structures [1-4]. The use of nanomaterials, which replace the rigid backbone of the naturally occurring toxins, may prove to be a fruitful approach for such an endeavor. In the past, fullerenes suffered from high production costs which generated an obstacle to the development of fullerene-based applications, but the cost has rapidly declined [5].

## Conclusions

Voltage-gated sodium channels are present throughout muscle and neuronal cells in mammals. Their dysfunction has long been linked to disorders such as epilepsy and chronic pain. Toxins from venomous species such as cone snails and scorpions have demonstrated activity against sodium channels. One example is the polypeptide toxin μ-conotoxin (PIIIA), extracted from the cone snail, which has been shown to potently block both bacterial and mammalian Nav channels [16,17,52]. Unfortunately, converting toxins to drugs represents a considerable challenge [1-4]. We attempt to mimic the structure of μ-conotoxin by (1) replacing its bulky core with a C_84_ fullerene and (2) chemically attaching positively charged groups to the fullerene surface. Although fullerenes have previously been identified as possible ion channel blockers [10-15], no studies have demonstrated the potential of designing fullerenes through chemical modification to target specific ion channels. We demonstrate that [Lys]-fullerene blocks the ion-conducting pathway of the bacterial voltage-gated sodium channel, Na_v_Ab with a strong binding affinity, similar in magnitude to μ-conotoxin [16], and with selectivity over potassium voltage-gated channels. This fullerene design may serve as a structural template from which a new set of potent compounds can be designed for the treatment of various diseases linked to sodium channel dysfunction.

## Competing interests

The authors declare that they have no competing interests.

## Authors’ contributions

TAH conceived the study, participated in its design, conducted the simulations, and drafted the manuscript. S-HC conceived the study, participated in its design and analysis, and helped draft the manuscript. Both authors read and approved the final manuscript.
